# Water purification ultrafiltration membranes using nanofibers from unbleached and bleached rice straw

**DOI:** 10.1038/s41598-020-67909-3

**Published:** 2020-07-09

**Authors:** Mohammad L. Hassan, Shaimaa M. Fadel, Ragab E. Abouzeid, Wafaa S. Abou Elseoud, Enas A. Hassan, Linn Berglund, Kristiina Oksman

**Affiliations:** 1 Cellulose and Paper Department and Centre of Excellence for Advanced Sciences, National Research Centre, 33 El-Buhouth street, Dokki, 12622 Giza Egypt; 2 Department of Engineering Sciences and Mathematics, Luleå University of Technology, 97187 Luleå, SE Sweden; 3 Department of Mechanical and Industrial Engineering, University of Toronto, 5 King’s College Road, Toronto, ON M5S 3G8 Canada

**Keywords:** Nanoscale materials, Nanoscale materials, Polymers

## Abstract

There has been an increasing interest in recent years in isolating cellulose nanofibers from unbleached cellulose pulps for economic, environmental, and functional reasons. In the current work, cellulose nanofibers isolated from high-lignin unbleached neutral sulfite pulp were compared to those isolated from bleached rice straw pulp in making thin-film ultrafiltration membranes by vacuum filtration on hardened filter paper. The prepared membranes were characterized in terms of their microscopic structure, hydrophilicity, pure water flux, protein fouling, and ability to remove lime nanoparticles and purify papermaking wastewater effluent. Using cellulose nanofibers isolated from unbleached pulp facilitated the formation of a thin-film membrane (with a shorter filtration time for thin-film formation) and resulted in higher water flux than that obtained using nanofibers isolated from bleached fibers, without sacrificing its ability to remove the different pollutants.

## Introduction

Membranes are used in a wide variety of industries for removing undesirable materials from different media. Among them, those that depend on separation based on the size of materials to be removed, e.g., micro-, ultra- and nano-filtration membranes, are manufactured using different polymers; generating pores within these membranes with targeted size is the key for their use^1^.

Using cellulose and its derivatives for making membranes for use in micro- and ultrafiltration is well known at the commercial level^2^. Cellulosic membranes are usually prepared using casting technique, which depends on the dissolution of cellulosic materials in suitable solvents, followed by film formation by immersion in a non-solvent. This technique requires use of large amounts of solvents; their recovery is important for economic and environmental reasons.

Since the emergence of nanocellulosic materials, e.g., cellulose nanofibers and cellulose nanocrystals, increasing research is ongoing regarding their use in the area of membranes. Two approaches have been investigated for use of nanocellulose in that area so far^3^. The first approach is through their incorporation in other polymer matrices to improve the performance of prepared membranes. Dissolving the polymers in suitable solvents and also good dispersion of nanocellulosic materials in the polymer solution are necessary before film casting. The second approach studied, which is more favorable and interesting, is to make membranes from a layer of nanocellulose with adequate porosity over other polymeric supports without the need to dissolve the cellulose and to use the film casting technique to generate porosity. The second approach was first introduced by Ma et al.^4^; it depends on the correlations between pore size distribution of a nonwoven layer structure and bulk porosity. According to this approach, the pore size of a film made from fibers can be adjusted by controlling the fiber diameter. It was also proved through a study of the pore size of films made from electrospun nanofibers that the mean pore size was found to be a factor of ~ 3 ± 1 greater than the mean fiber diameter and that the maximum pore size was a factor of ~ 10 ± 2 greater than the mean fiber diameter^4^. Because cellulose nanofibers and nanocrystals have diameters within a few nanometers, generally from 4 to < 100 nm, formation of membranes suitable for ultra- and nanofiltration purposes could be expected. Cellulose nanofibers can be isolated from cellulose pulp with much higher yield because their isolation depends on applying a high mechanical shear force onto the pulp fibers. Cellulose nanocrystals are prepared by acid hydrolysis of pulps using a relatively high concentration of acids, and the yield of the isolated nanocrystals is relatively low.

The use of cellulose nanofibers has been investigated much more than that of cellulose nanocrystals to make films for ultrafiltration membranes because the nanofibers have much better ability to form thin films with good flexibility and less aggregation than the nanocrystals^5–8^. The published works so far regarding making films from cellulose nanocrystals for ultrafiltration applications have focused on their use as self-standing nanopaper sheets^5^, thin films over cellulose nanofibers^6^, thin films over filter paper and comparing them to cellulose nanofibers^7^, and thin-layer films over polyethersulfone and comparing them to cellulose nanofibers^8^. However, cellulose nanofibers were used for making thin-film membranes in more studies^3,9–11^, in addition to their use as additives in other polymeric matrices to improve the properties of prepared membranes^3,12^.

The literature survey mentioned above reveals that cellulose nanofibers used in making membranes were extracted from bleached cellulose pulp fibers, i.e., fibers mostly from cellulose and some hemicelluloses but without or with very little lignin^3,9–11^. A common challenge associated with membrane preparation from cellulose nanofibers is formation of compact films of these nanofibers upon drying as a result of extensive hydrogen bonding between hydroxyl groups at their surfaces. This results in low-flux membranes, especially when filtering suspensions containing particles with small size or high-molecular-weight materials. To overcome this problem, researchers have studied different solutions, such as exchanging water in the wet nanofiber film after formation of membranes by using other solvents such as alcohol^10,13^. This resulted in formation of less dense films and improving the porosity and thus the flux of the membranes. Others studied the addition of other particles to the nanofiber suspension before membrane preparation and drying; this added gaps between the nanofibers and thus improved the porosity and flux^10,14^. Others studied chemical modification of nanofiber’ surfaces by spacers. Upon membrane formation, these spacers act as gaps between the nanofibers, leading to improved water flux of the membranes^11^. All of the aforementioned solutions definitely add to the cost of making membranes with good flux and rejection.

The current work focuses on the use of cellulose nanofibers isolated from unbleached pulp, i.e., nanofibers with high lignin content, to make membranes with good flux and rejection. Isolation of cellulose nanofibers from unbleached pulp recently found increasing attention not only for producing cellulose nanofibers with lower cost and environmental impact from eliminating bleaching but also for producing nanofibers with different surface properties. The presence of lignin at the surface of the nanofibers is expected to reduce the extensive hydrogen bonding between the nanofibers and thus membranes with higher porosity and flux can be prepared without the need to exchange water, to add other particles, or to chemically modify the surface.

## Results and discussion

### RSNF isolated from rice straw pulps

**Figure 1:**
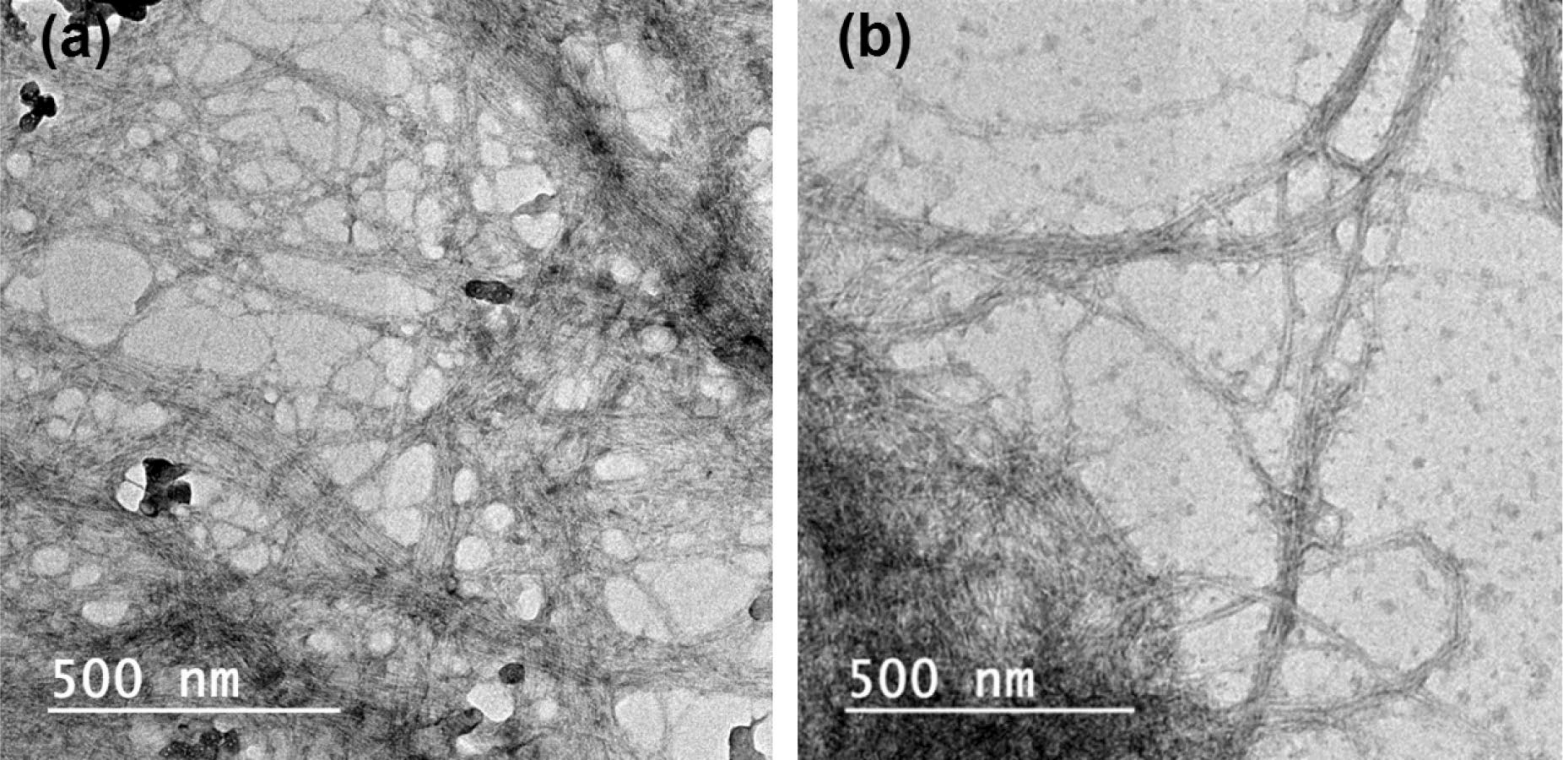
TEM images of RSNF isolated from (**a**) unbleached neutral sulfite pulp and (**b**) bleached soda pulp.

Neutral sulfite pulp is a kind of high-yield pulp characterized by relatively high lignin content; the pulp is generally used in paperboard manufacturing. During pulping, a small amount of lignin originally present in the lignocellulosic materials is dissolved while the rest is slightly modified and retained within the fibers. In a previous publication^15^, we showed the possibility of isolating RSNF with diameters close to that of elementary fibrils (4–7 nm in width) from unbleached neutral sulfite rice straw pulp with high lignin content by ultrafine grinding. Figure 1 shows TEM images of RSNF isolated from unbleached neutral sulfite pulp and bleached soda pulp. Both nanofibers have similar diameters (~ 4–7 nm) while their lengths were several micrometers.

### Membranes prepared from RSNF

**Figure 2:**
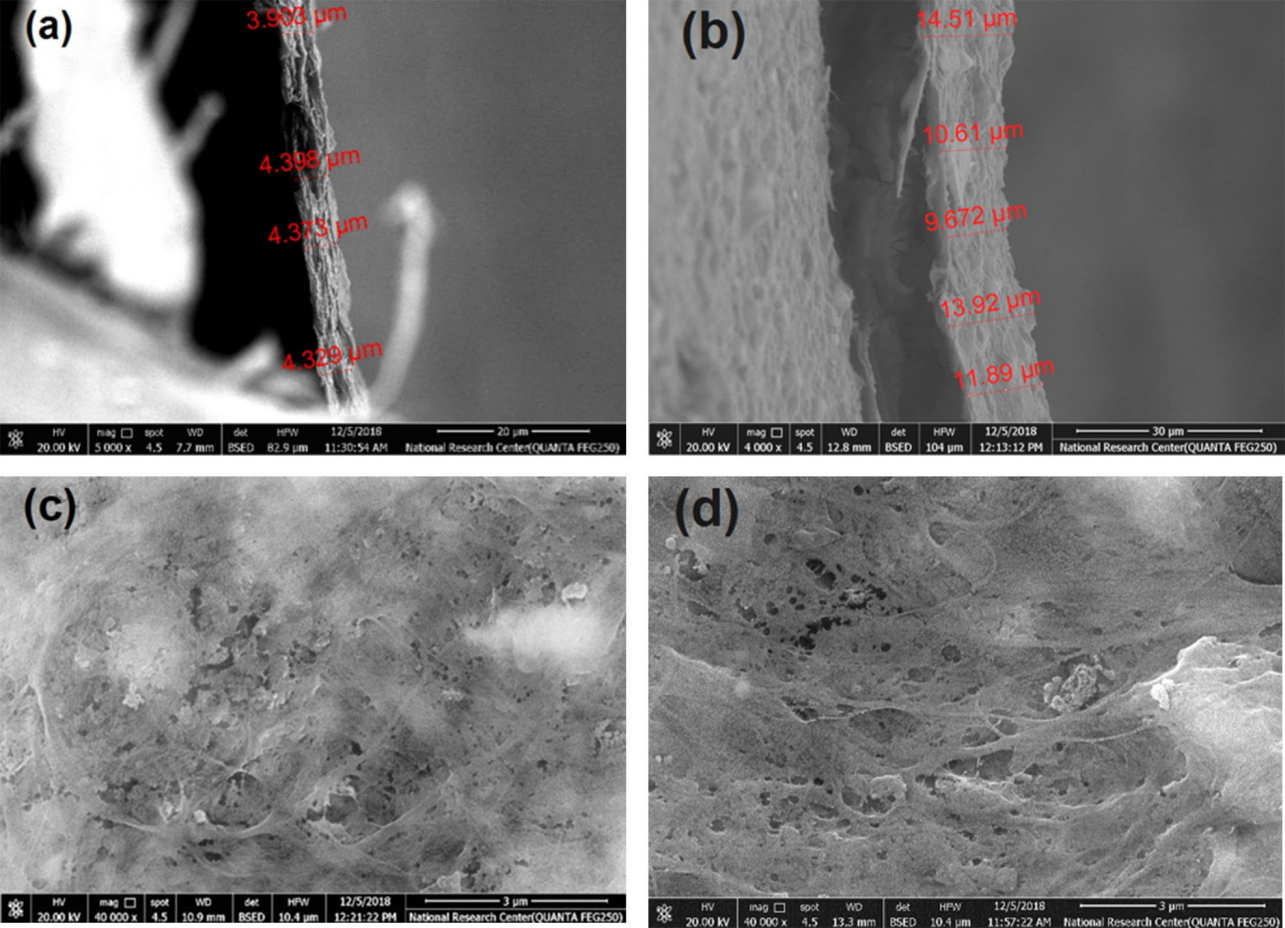
Scanning electron microscope images of the cross sections and surfaces of (**a**,**c**) bleached RSNF membrane and (**b**,**d**) unbleached RSNF membrane.

Membranes from RSNF isolated from unbleached and bleached pulps were prepared by vacuum filtration over hardened filter paper; PAE was used as a cross-linker. It was noticed during vacuum filtration that formation of films over hardened filter paper was much faster when using RSNF isolated from the unbleached pulp fibers. Figure 2 shows cross sections and surfaces of membranes formed from equal weights of the nanofibers isolated from bleached and unbleached pulps on filter paper (hereafter referred to as bleached RSNF and unbleached RSNF membranes, respectively). The formed membrane films had a basis weight of ~ 2 g/m^2^. As shown in the figure, the thickness of the film formed from bleached RSNF was ~ 4 μm while that of unbleached RSNF was ~ 11 μm. This indicates that, upon drying the unbleached RSNF membrane, much less hydrogen bonding took place between the nanofibers, resulting in a thicker film.

However, the surface of the prepared membranes exhibited no significant difference in pore diameters. The imaged parts exhibited pore diameters of 128 ± 24 and 106 ± 28 nm for membranes prepared from bleached and unbleached RSNF membranes, respectively. The diameter of the pores at the surface qualifies the prepared membranes for use in ultrafiltration.

To further prove the higher porosity of the membrane formed from unbleached RSNF, surface area measurements were conducted on self-standing membranes prepared from unbleached and bleached RSNF. The BET nitrogen adsorption curves are given in Supplementary Fig. S1 online. Surface area, pore volume, and pore diameter of RSNF unbleached membrane were 507 m^2^/g, 0.89 cm^3^/g, and 1.92 nm, respectively, while that of RSNF bleached membrane were 240 m^2^/g, 0.42 cm^3^/g, and 1.92 nm, respectively. The results clearly show that the unbleached RSNF membrane has a 111% higher surface area and a 112% higher pore volume than that of the bleached RSNF membrane.

The hydrophilicity of the surfaces of RSNF membranes was investigated by measuring the water contact angle (Supplementary Fig. S2 online). As shown in the figure, the surface of the bleached RSNF membrane is more hydrophilic than that of the unbleached RSNF membrane since water contact angle was about 49° and 85° for the former and later kind of membranes, respectively. This could be attributed to the presence of more hydrophilic groups on the surface of bleached nanofibers (hydroxyl and carboxylic groups) than in nanofibers isolated from unbleached pulp because of the presence of lignin (poly phenolic polymer).

### Pure water flux and fouling of RSNF membranes

**Figure 3:**
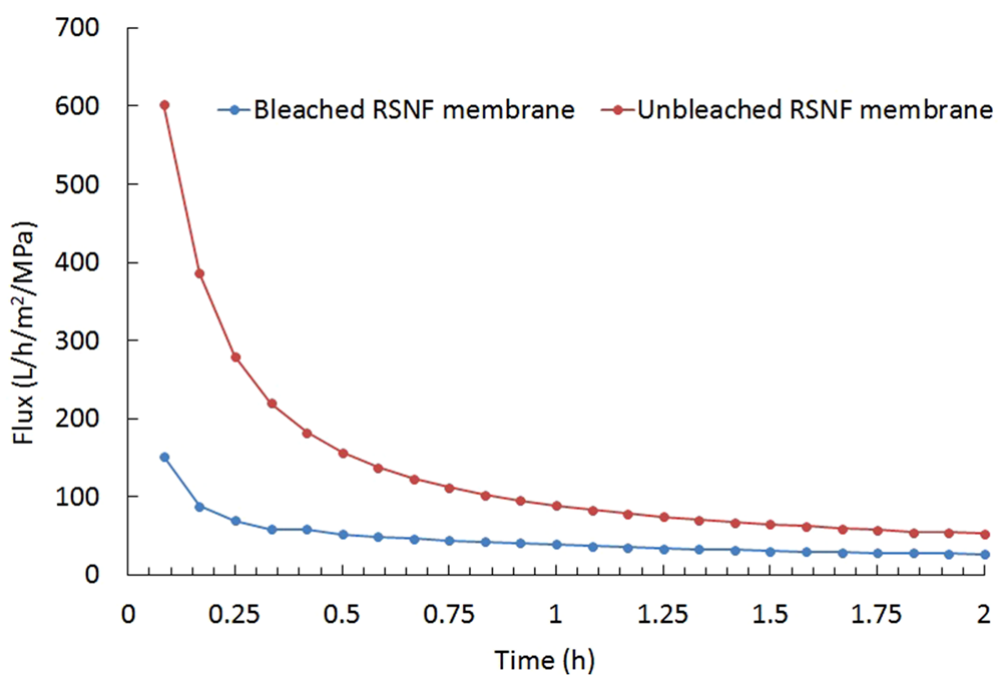
Pure water flux of unbleached and bleached RSNF membranes.

Figure 3 shows the pure water flux of unbleached and bleached RSNF membranes. As is clear from the figure, the unbleached RSNF membrane had higher water flux than the bleached RSNF membrane. This could be due to the higher porosity and pore volume of the unbleached RSNF membrane. Based on the contact angle and surface area measurements mentioned above, the water flux results mean that the effect of porosity and pore volume of the membranes exceeded that of hydrophilicity at the surface. The results in Fig. 3 show a decrease in water flux with increasing time of filtration owing to the compaction of the membranes by the pressure applied^5,17^. The presence of lignin, a three-dimensional phenolic polymer^18^, at the surface of the RNSF isolated from unbleached pulp could make the nanofibers less affected by compaction than those without lignin, e.g., isolated from bleached pulp. After the experiment was run for 2 h, the water fluxes were ~ 27 and ~ 53 L/h/m^2^/MPa for bleached and unbleached RSNF membranes, respectively; i.e., the water flux value was 96% higher in the case of the unbleached RSNF.

The improvement of water flux in the current work resulting from using unbleached nanofibers instead of bleached nanofibers was compared to that reported in a previous work where bleached nanofibers were exchanged with alcohol before drying for making membranes^10^. A ~ 200% improvement in water flux was achieved after passing water for 1 h for membranes exchanged with alcohol, whereas in the current work, the improvement in flux as a result of using unbleached nanofibers instead of bleached ones was ~ 120% after the same flux time. Exchanging water with alcohol before making membranes from the unbleached nanofibers used in the current work could also lead to a further increase in porosity owing to the less compact structure and thus higher water flux.

**Figure 4:**
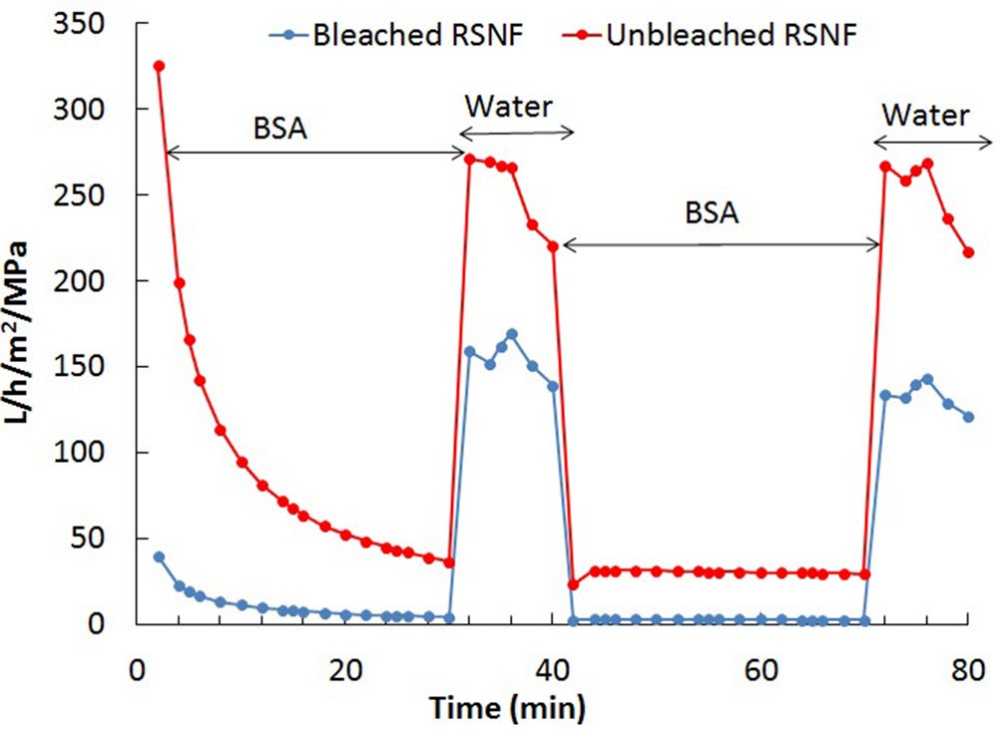
Flux cycles of BSA solution and water for membranes prepared from RSNF isolated from bleached and unbleached pulp.

Figure 4 shows the effect of cycles of passing BSA aqueous solution and pure water across the membranes to investigate membrane fouling. As shown in the figure, after 30 min of passing the BSA solution (the first BSA cycle), the water flux value for the bleached RSNF membrane was 4.4 L/h/m^2^/MPa, whereas that for the unbleached RSNF membrane was 36.5 L/h/m^2^/MPa. At the end of the second BSA cycle, these water flux values were 2.6 and 27 L/h/m^2^/MPa for bleached and unbleached RSNF membranes, respectively. These results indicate that the higher porosity and pore volume of the unbleached RSNF membrane helped keeping the membrane structure open enough and less susceptible to clogging by BSA molecules. During the pure water cycles, washing the surface of the membranes with water removed the BSA accumulated at the surface, and water passed through the membrane washed away BSA in the pores, resulting in increased water flux**.** For both bleached and unbleached RSNF membranes, no significant drop of pure water flux value occurred after the second BSA cycle.

**Figure 5:**
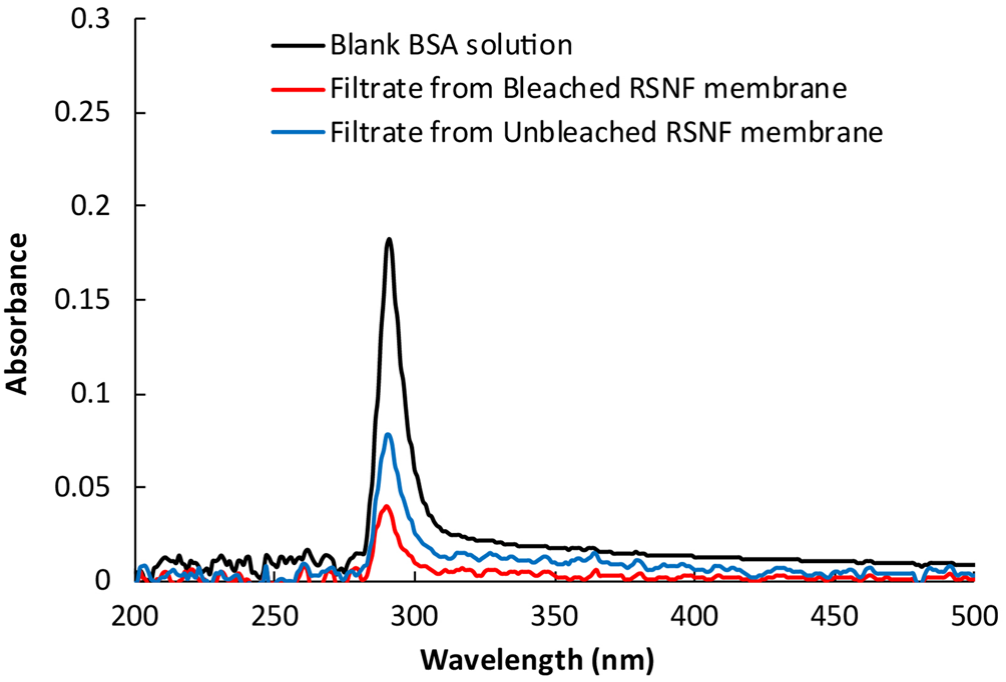
UV absorbance curves of BSA filtrate from bleached and unbleached RSNF membranes.

Figure 5 shows the UV–visible absorbance spectra of BSA filtrates from the bleached and unbleached RSNF membranes. According to the absorbance values of the filtrates and the standard curves for BSA solutions of different concentrations (Supplementary Fig. S3 online), the unbleached RSNF membrane could reject 58.4% of the BSA soluble in water, whereas the bleached RSNF could reject 79.7% of the BSA. The higher rejection of BSA from water by the bleached RSNF membrane could be due to its much lower porosity and more compact structure compared to that of the unbleached RSNF membrane.

### Use of RSNF membranes for removal of lime nanoparticles

Unbleached and bleached RSNF membranes were tested to remove lime nanoparticles as a model for sub-micrometer particles; their average diameter was 477 ± 264 nm as determined by laser particle size analysis and observed in TEM images (Supplementary Fig. S4 online).The cumulative particle size distribution of the lime nanoparticles as determined by laser is shown in Supplementary Table S1.

**Figure 6:**
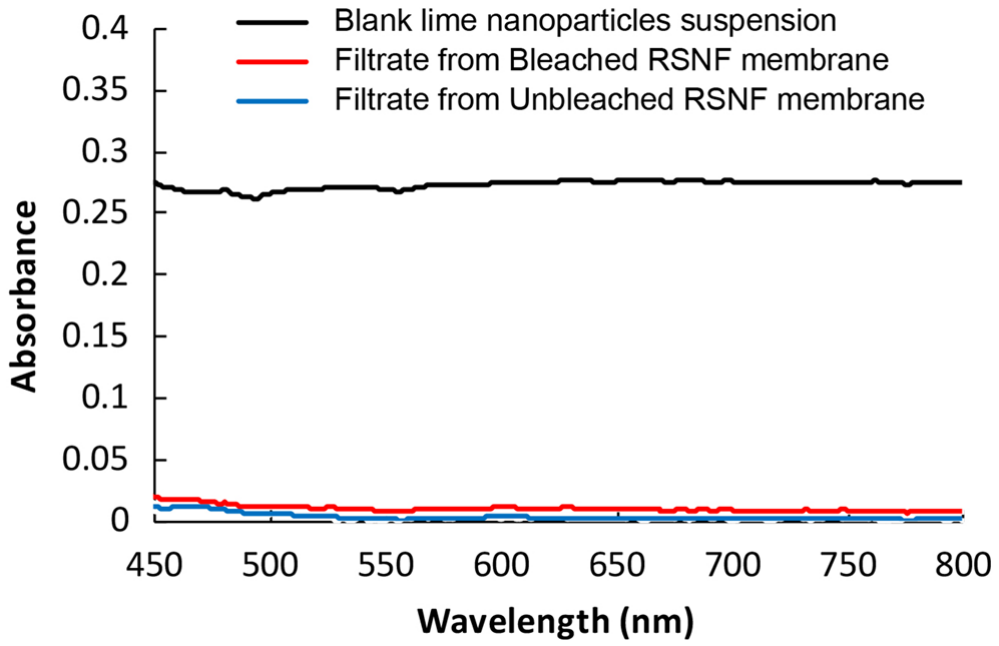
Visible spectra of lime nanoparticle filtrate from the RSNF membranes.

The efficiency of removing lime nanoparticles was followed by measuring the turbidity of the lime suspension before and after filtration. The measurement is based on the amount of light scattered by the nanoparticles suspended in white water. Figure 6 shows the absorbance spectra in visible light of a blank lime nanoparticles suspension and the filtrate after passing through RSNF membranes. A remarkable decrease in turbidity took place upon filtration of the nanoparticle suspension by the different membranes. According to the turbidity standard curve of lime nanoparticles suspensions (Supplementary Fig. S5 online), the estimated removal of lime nanoparticles was ~ 99% and ~ 97% by bleached and unbleached RSNF membranes, respectively.

After passing 250 mL of a 1% lime nanoparticle suspension across RSNF membranes, the fluxes recorded were 89.7 ± 1.5 and 92.8 ± 2.2 L/h/m^2^/MPa for unbleached and bleached RSNF, respectively.

### Use of RSNF membranes for purification of white water

White water produced from paper mills working on recycled fibers contains a considerable amount of suspended particles with different diameters, ranging from hundreds of micrometers to even less than a micrometer, as a result of the presence of fillers and colloidal materials from latex binders, dispersants from recycled coated grades, polyelectrolyte complexes formed, and other papermaking additives^19,20^. Because white water is reused multiple times in a modern paper machine system, purification of white water is an essential step before reuse to avoid the negative effects of these contaminants on the properties of produced paper products or during production. Use of ultrafiltration is one of the practiced technologies for treatment of white water because it allows removal of very fine particles (solid and colloidal particles) with sizes in the sub-micrometer level^21^.

In the current work, the efficiency of RSNF membranes in removal of fines and colloidal particles from papermaking white water was estimated by measuring the turbidity before and after filtration through the membranes. The average particle size of suspended particles remaining in the filtrate was 1.74 ± 1.98 µm; Supplementary Fig. S6 shows the particle size distribution of white water contaminants after being filtered by vacuum through filter paper to remove the relatively large particles. Supplementary Table S2 lists the cumulative distribution of the particle size for these suspended particles; the results in the table show that 25% of the particles have diameters of < 530 nm, 25% of the particles have diameters from 530 nm to ~ 1 µm, 25% of the particles have diameters from 1 to 2.1 µm, 5% have diameters from 2.1 to 2.5 µm, 10% have diameters from 2.5 to 4 µm, and 10% have diameters from 4 to 11 µm.

**Figure 7:**
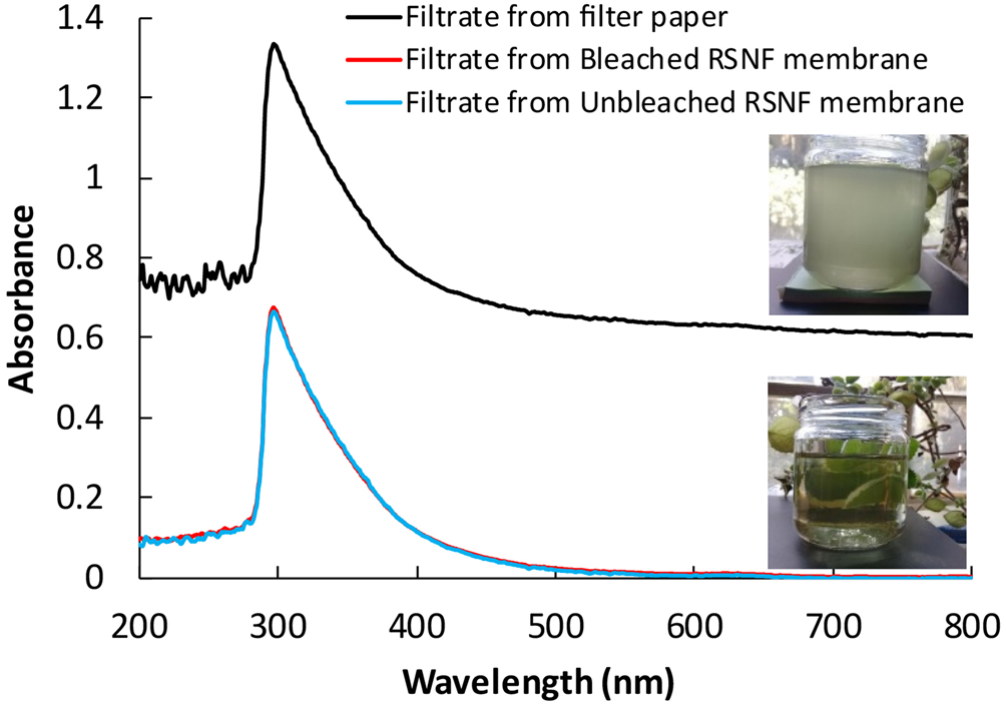
UV–visible absorbance spectra of white water after filtration through filter paper and through unbleached and bleached RSNF membranes.

Figure 7 shows the UV-visible spectra of white water before and after passing through the different RSNF membranes. Based on turbidity values at 600 nm, almost complete removal of the suspended particles could be achieved using bleached and unbleached RSNF membranes. Photographs of the white water before and after filtration through the membranes clearly show the ability of the membranes to remove the suspended particles. In addition to removing suspended particles, RSNF membranes can also partially remove other soluble organic materials from white water, as is clear from the decrease of the peak at 295 nm by ~ 50% after passing through the different membranes.

**Figure 8:**
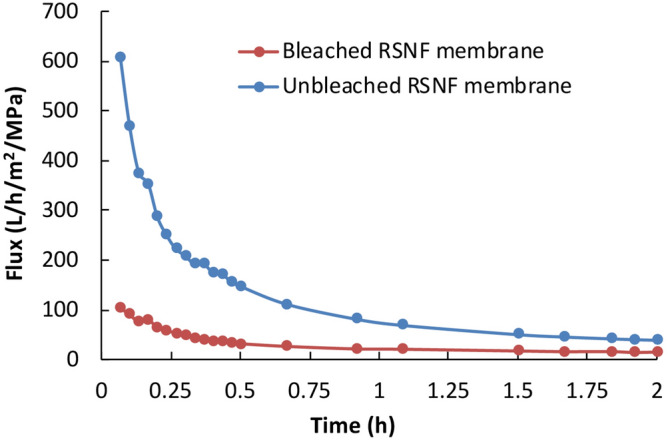
White water flux of bleached and unbleached RSNF membranes.

Figure 8 shows flux of white water through the membranes. The curves clearly show the advantage of using the unbleached RSNF membrane over the bleached RSNF one. After ~ 2 h of filtration, the flux values were ~ 40 and ~ 15 L/h/m^2^/MPa for unbleached and bleached membranes, respectively. The higher flux of the former membrane could be attributed to its higher porosity and pore volume, as mentioned before.

## Conclusions

Cellulose nanofibers isolated from unbleached rice straw neutral sulfite pulp can be used instead of those isolated from bleached pulp to make membranes with lower cost, higher water flux, less tendency to fouling, and good ability to reject the different size particulates used in the current study, e.g., lime nanoparticles and sub-micrometer fines in white water from papermaking. Using nanofibers from unbleached pulp in making membranes can effectively decrease the compactness of the nanofibers upon drying and result in membranes with higher surface area and higher average pore volume, providing higher water flux than in the case of using nanofibers isolated from bleached pulp. From the results of water contact angle and porosity in the current work, it can be concluded that the porosity of membranes (surface area and pore volume) was more effective in increasing water flux than the hydrophilicity of membranes.

## Materials and methods

### Materials

Rice straw raw material was collected from local farms in Giza, Egypt. The straw was washed with tap water to remove dirt and was left to dry in air. Sodium carbonate sodium sulfite, sodium chlorite (technical grade 80%), and glacial acetic acid were reagent grade chemicals and used as received from Fisher Scientific (Loughborough, UK). Polyamide-amine-epichlorohydrin (PAE) was a commercial grade solution with 33 wt.% solid content (~ 33%, w/w solid content, Solenis, Wilmington, Delaware, USA). PAE solution was diluted to 1 wt.% with distilled water prior to use. Lyophilized bovine serum albumin (BSA) was purchased from the Biowest Company (Naillé, France) and was used as received.

### Preparation of rice straw pulp

Unbleached neutral sulfite pulp was prepared as previously described by Hassan et al.^15^. In brief, rice straw was treated with an aqueous solution containing 10% sodium sulfite and 2% sodium carbonate aqueous (based on rice straw weight) at 160 °C for 2 h and the liquor ratio was 1:10. The pulp was thoroughly washed with water, defibrillated using a Valley beater (Valley Iron Works, Appleton, Wisconsin, USA) to 25°SR degree of freeness. The pulp was then dewatered and left to dry in air. The chemical composition of the produced pulp was 14.15% Klason lignin, 54.12% α-cellulose, 14.34% pentosans, 16.63% ash content, and degree of polymerization 903.

The produced pulp was bleached according to the previously published method using a sodium chlorite–acetic acid mixture at 80 °C for 1 hour^16^. The chemical composition of the bleached pulp was 1.32% Klason lignin, 68.4% α-cellulose, 18.6% pentosans, 12.47% ash content, and degree of polymerization 834.

### Isolation and characterization of rice straw nanofibers

Rice straw nanofibers (RSNF) from rice straw bleached and unbleached pulps were isolated according to the previously published protocol by Hassan et al.^15^. The pulps were first disintegrated by a shear mixer (Silverson L4RT, Silverson Machines Ltd., Chesham, UK) using 2 wt.% consistency pulp suspensions. The disintegrated pulps were then fibrillated by a high-shear ultrafine friction grinder (MKCA6-2, Masuko Sangyo, Kawaguchi, Japan). The disks of the grinder were gradually adjusted to − 90 µm; the pulps were run through the grinder for ~ 140 min.

Transmission electron microscopy (TEM) was conducted using a high-resolution JEM-2100 transmission electron microscope (JEOL, Tokyo, Japan), and an acceleration voltage of 100 kV was used in the examination. The fiber suspension was dropped on a copper grid bearing a carbon film and left to dry before examination.

### Papermaking white water

In the papermaking industry, the term “white water” refers to the aqueous suspension that drains from wet paper sheet formation. White water was kindly supplied by the United Company for Paperboard, Sadat City, Egypt. White water was collected from papermaking machine drainage of paperboard made from recycled fibers. The white water was first left to settle the large particles, which were separated by decantation. Then, it was filtered through hardened filter paper (F2041 grade, CHMLAB, Barcelona, Spain) to remove microparticles. The particle size distribution of suspended particles in white water after filtration was examined using a zetasizer instrument (Zetasizernano, Malvern Panalytical, Malvern, UK). Total dissolved solids and conductivity were measured with a conductivity meter (Jenway 4510, Staffordshire, UK). The solid content of the filtered white water was 0.16 wt.%, its pH was 7.8, its conductivity was 2.41 mS/cm, and total dissolved solids was 1.42 g/L.

### Preparation and characterization of lime nanoparticles suspension

Lime nanoparticles were prepared using 0.3 mol/L calcium hydroxide and 0.6 mol/L sodium hydroxide aqueous solutions as previously published^12^. In brief, sodium hydroxide solution was added dropwise to calcium chloride solution at a rate of ~ 1 mL/min at 90 °C. The precipitated lime nanoparticles were washed with previously boiled distilled water by repeated centrifugation at 10,000 rpm. The purified lime nanoparticle suspension was kept in a closed bottle flushed with nitrogen until use. TEM and particle size analysis by laser were conducted as mentioned above.

### RSNF membrane preparation

Membranes from the nanofibers were prepared by vacuum filtration on 9-cm hardened filter paper (F2041 grade, CHMLAB, Barcelona, Spain) using a vacuum pump as previously described^9^. The nanofiber water suspension (0.1 wt% consistency) containing PAE cross-linker at 2 wt.% (based on the oven-dry weight of nanofibers) was filtered on the filter paper and oven-dried at 80 °C for 1 h to achieve cross-linking between the nanofibers by the added PAE.

### Evaluation of membrane properties

Microscopic features of the membranes were examined using a FEI Quanta 200 scanning electron microscope (FEI Company, Eindhoven, The Netherlands); an acceleration voltage of 20 kV was used. Samples were coated with gold before scanning in a sputter coater (Edwards sputter coater, Sussex, UK).

The Brunauer–Emmett–Teller (BET) surface area, pore volume, and average pore radius of membranes were measured by using a Quantachrome Nova-1200 instrument (Quantachrome Instruments, Florida, USA). The membranes were degassed prior to measurement at 100 °C for 12 h. The membranes used in the test were prepared by vacuum filtration, as mentioned in Sect. 2.7, but on nitrocellulose disks to be able to separate dried membranes.

The water contact angle of membranes was measured by using an Attension Theta Lite measuring system (Biolin Scientific AB, Gothenburg, Sweden). The contact angle was calculated with the drop shape analysis software One Attension Version 2.7, using a sessile drop technique. Water drops of 4 μL in size were placed at the surface of membranes at four separate places, and the average contact angle was calculated.

The pure water flux of membranes was measured according to the previously published method by Hassan et al.^9^ using a dead-end stirred cell (Sterlitech HP4750, Kent, Washington, USA) at 25 °C and a differential pressure of 20 MPa maintained using hydrolytic pump; the active filtration area was 14.6 cm^2^. Disks of 5 cm in diameter were cut out from the membranes and soaked in water for 1 h before measurement. The water flux *J* in L/(h/m^2^/MPa) was calculated from accurately weighing the quantity of water passing through the membranes for a defined time interval using Eq. (1):

1 $$J = v/(atp).$$

where *v* is the volume of the permeate (in liters), *a* is the membrane area (in m^2^), *t* is time (in hours), and *p* is the pressure used in the test (in MPa).

Fouling of the membranes was tested using a BSA solution (1 g/L). The solution was passed through the membranes for 30 min under a pressure of 30 MPa in the dead-end stirred cell and the flux was calculated. Then, the membranes were briefly washed with distilled water and pure water was passed through the membranes under 30 MPa pressure for 10 min, and flux was calculated. This cycle was repeated twice, and the test was replicated to calculate the average flux values.

Membranes prepared from nanofibers isolated from unbleached and bleached pulps were tested for removing BSA, the laboratory-prepared lime nanoparticles, and fine particulates from papermaking white water. For the BSA and lime nanoparticles, a solution or suspension containing 1 g/L was passed through the membrane using the dead-end stirred cell at 25 °C and a differential pressure of 30 MPa maintained using hydrolytic water pump; the active filtration area was 14.6 cm^2^. For BSA, absorbance at 291 nm was measured before and after passing through the membranes using a Jenway 7205 UV–visible spectrometer (Staffordshire, UK). A standard curve of concentration versus absorbance at 291 nm was set for a series of BSA solutions with known concentrations. The concentration of BSA in the filtrate was calculated from a standard curve equation. Rejection efficiency was calculated using Eq. (2):

2 $${\rm{rejection }}\left( \% \right) \, = \, \left[ {\left( {{\rm{control}}A_{{{291}}} - {\rm{ sample}}A_{{{291}}} } \right)/{\rm{control}}A_{{{291}}} } \right] \, \times 100.$$

For lime nanoparticles, the turbidity of the suspension before and after filtration was calculated from measuring the absorbance of light at 600 nm using a Jenway 7,205 UV–visible spectrometer (Staffordshire, UK) as follows: turbidity = 2.302 *A*/*l*, where *A* is the absorbance and *l* is the path length (0.01 m). A standard curve of concentration versus absorbance was set for a series of lime nanoparticle suspensions with known concentrations. The concentration of lime nanoparticles in the filtrate was calculated from a standard curve equation. Rejection efficiency was calculated using Eq. (3):

3 $${\rm{Rejection }}\left( \% \right) \, = \, \left[ {\left( {{\rm{control}}T_{{{6}00}} - {\rm{ sample}}T_{{{6}00}} } \right)/{\rm{control}}T_{{{6}00}} } \right] \, \times 100$$

For papermaking white water, the wastewater was passed first through hardened filter paper (F2041 grade, CHMLAB, Barcelona, Spain) to remove large particles, and then the filtrate was passed through the dead-end stirred cell at 25 °C. A differential pressure of 5 MPa was maintained using nitrogen gas, and the active filtration area was 14.6 cm^2^. The turbidity at 600 nm of white water before and after filtration through the membranes was measured, and rejection was calculated as mentioned above.

## Supplementary information

Supplementary information.

## Data Availability

All data generated or analyzed during this study are included in this published article (and its Supplementary Information files).
