# Supplementary material for: Water purification ultrafiltration membranes using nanofibers from unbleached and bleached rice straw
Source: Sci Rep. 2020 Jul 9;10:11278. doi: 10.1038/s41598-020-67909-3 (PMC7347555; doi:10.1038/s41598-020-67909-3)
Supplement: Supplementary file 1 — Supplementary information. [file 41598_2020_67909_MOESM1_ESM.pdf]

Water purification ultrafiltration membranes using nanofibers from unbleached and bleached rice straw.

Mohammad L. Hassan, Shaimaa M. Fadel, Ragab E. Abouzeid, Wafaa S. Abou Elseoud, Enas Hassan, Linn Berglund, Kristiina Oksman.

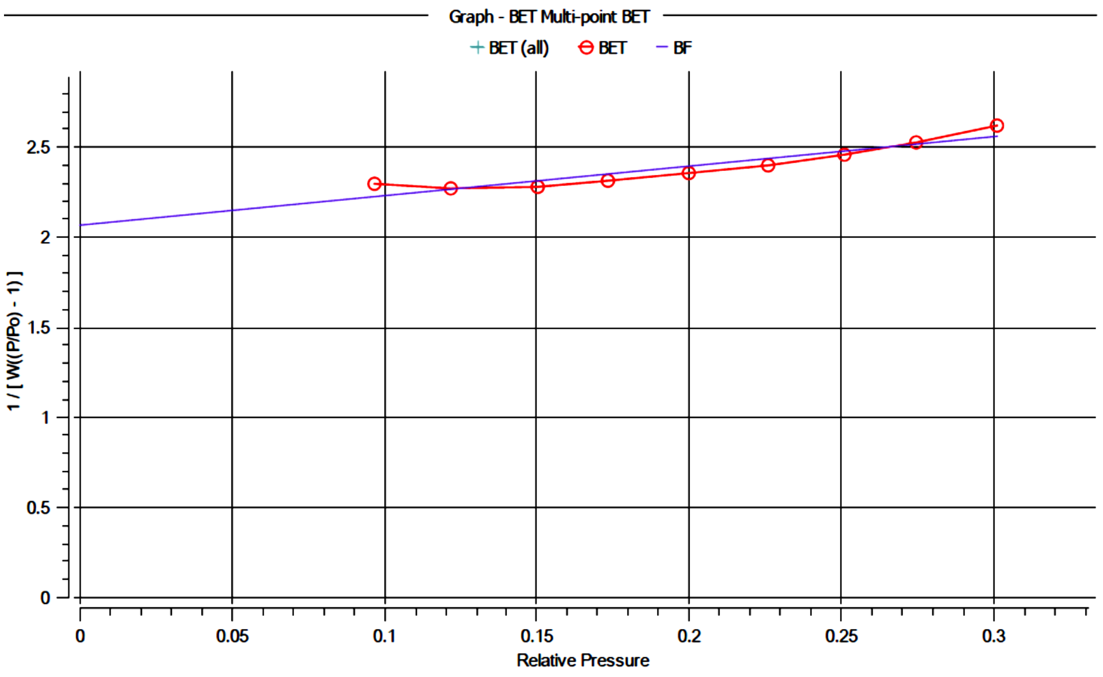

(a)

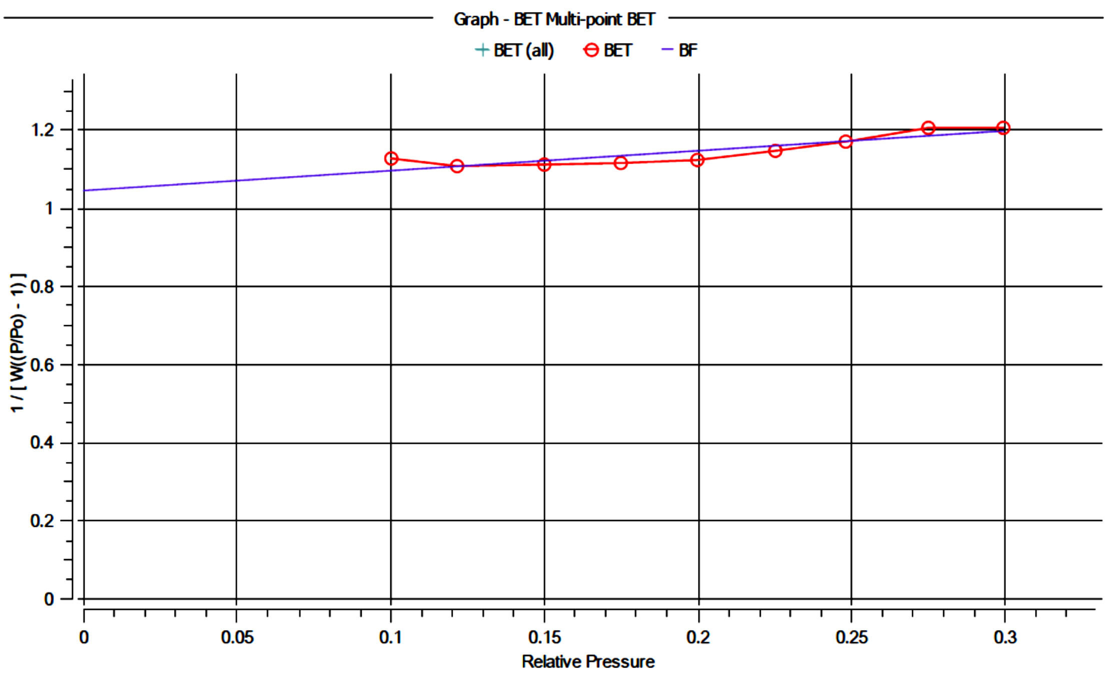

(b)

Supplementary Figure S1. BET isotherms for (**a**) bleached and (**b**) unbleached RSNF membranes.

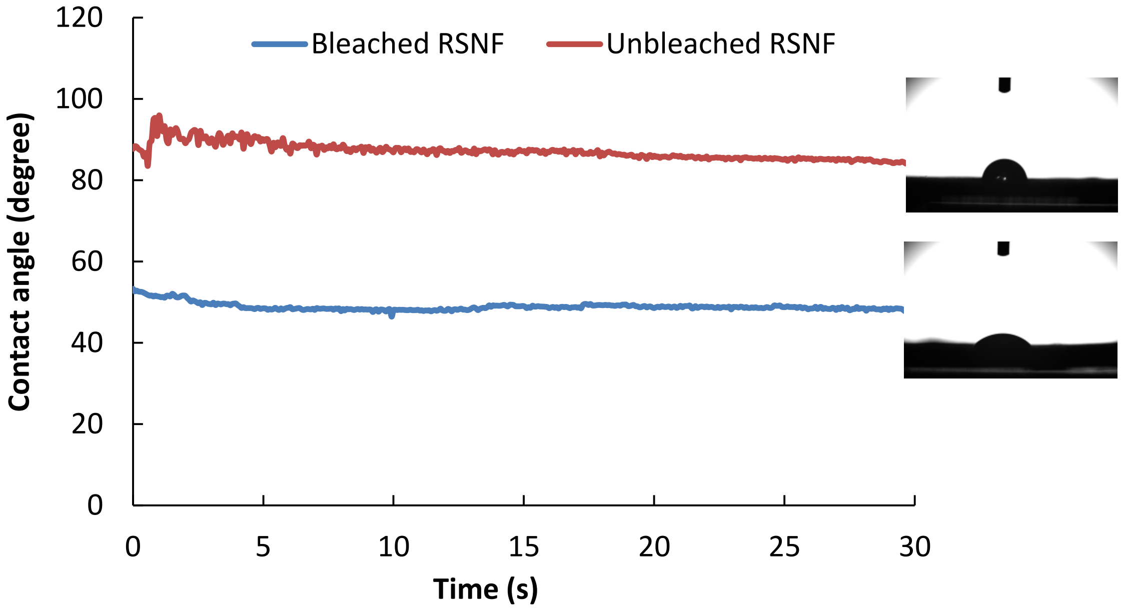

Supplementary Figure S2. Time versus water contact angle of unbleached and bleached RNSF membranes.

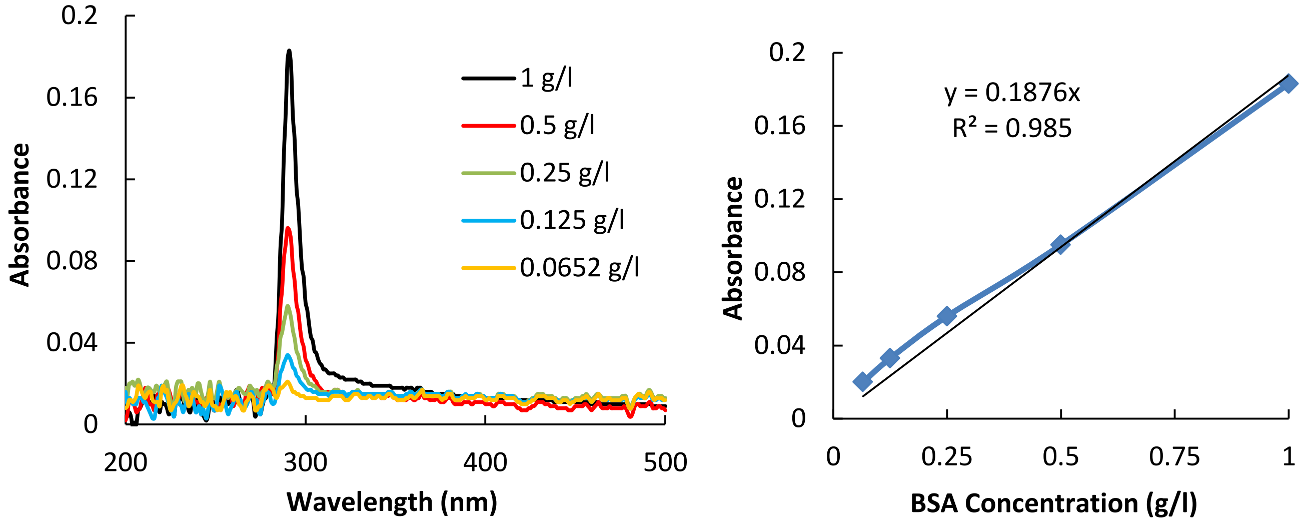

(a) (b)

Supplementary Figure S3. UV absorbance curves of BSA aqueous solution with different concentrations (**a**) and calibration curve (**b**).

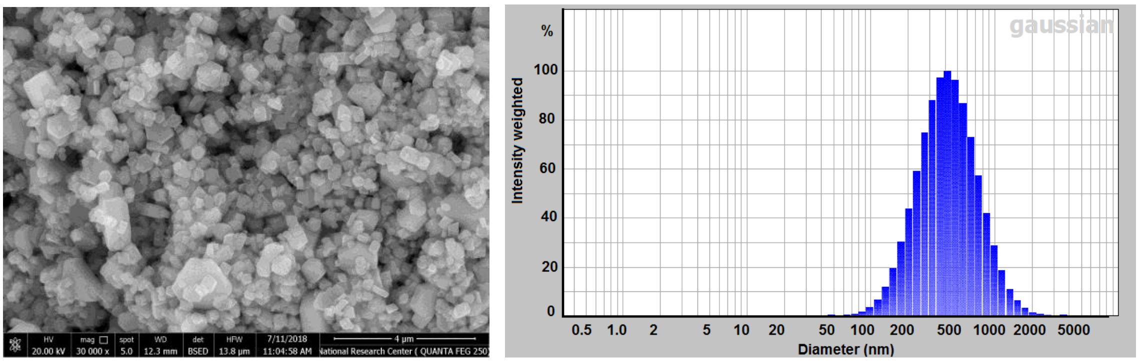

Supplementary Figure S4. TEM images of lime nanoparticles and their particle size distribution.

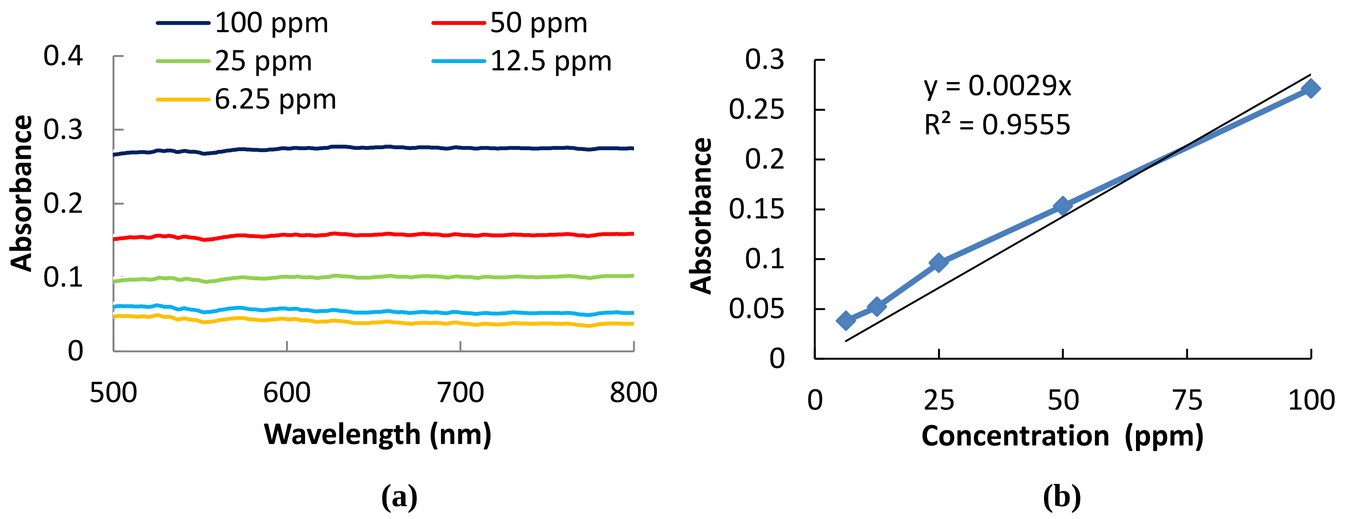

Supplementary Figure S5. Visible light absorbance curves of lime nanoparticles suspensions with different concentrations (**a**) and calibration curve (**b**).

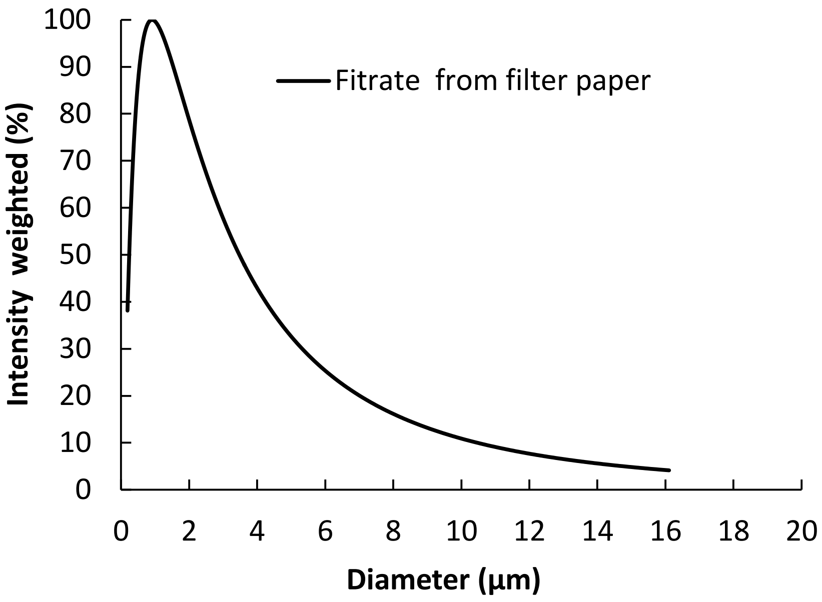

Supplementary Figure S6. Particle size distribution of white water contaminants after filtration through filter paper.

Supplementary Table S1. Cumulative results of particle size analysis of lime nanoparticles.

**% of distribution**	**Diameter (nm)**
25	<282
50	<409
75	<594
80	<652
90	<831
99	<1481

Supplementary Table S2. Cumulative results of particle size analysis of white water after filtration through filter paper.

**% of distribution**	**Diameter (μm)**
25	< 0.53
50	< 1.03
75	< 2.10
80	< 2.51
90	< 4.04
99	< 11.2
